# Mapping climate change-driven epidemics

**DOI:** 10.3389/fepid.2025.1605058

**Published:** 2025-07-29

**Authors:** Allyson Murray, Anna Ignaszak

**Affiliations:** ^1^ Laboratory Electrochemistry and Functional Materials, Department of Chemistry, University of New Brunswick, Fredericton, NB, Canada; ^2^ Department of Chemistry, Brock University, St. Catharines, ON, Canada

**Keywords:** climate change, dengue, yellow fever, malaria, HIV, Monkeypox, influenza, global warming

## Abstract

The recent analysis by Mora and colleagues revealed that over 277 diseases can worsen due to climatic hazards resulting from greenhouse gas emissions. Specifically, more than 58% of known human diseases can be aggravated by climate change. Furthermore, there are over 1,000 pathways through which various climatic hazards have contributed to disease outbreaks, primarily due to the diversity of pathogens. This analysis also urges immediate action to address the root of the problem—reducing greenhouse gas (GHG) emissions. Numerous climatic hazards affect the incidence of human pathogenic diseases. Unfortunately, due to the complexity and multifaceted nature of the problem, there cannot be a single comprehensive solution to minimize climate-driven outbreaks. This study seeks to identify outbreaks of specific diseases categorized as epidemics, whose incidence is strongly correlated with global warming. The focus of this analysis is on (1) organizations responding to climate-related diseases to decelerate the incidence rates; (2) to call for a new disciplines in epidemiology that focuses exclusively on climate change-related prediction for future pandemics; (3) looking at the problem from the patient's point of view—how do non-medical/health professionals contribute to minimizing the spread of climate-related diseases?; (4) to analyze outbreaks vs. urbanization/pollution/increase in population density and public health policies; also (5) to verify the vaccination coverage vs. case reduction rate.

## 1 Introduction

### 1.1 Global warming

Global warming refers to a gradual rise in the Earth's average temperature, affecting the atmosphere, oceans, and land surfaces (1). This phenomenon is generally considered to have begun in the mid-1800s, when society started to industrialize. An initial collection of temperature gathered reveals signals of temperature rising in the 1950s. However, today's hypothesis traces the start of global warming back 120 years to 1830 (2).

The earliest known theories on climate change are attributed to French scientist Joseph Fourier, who first proposed the concept of the “greenhouse effect,” whereby the sun's rays bouncing off the Earth are trapped by gases, eventually leading to warmer temperatures (3). Over the next century and a half, climate change theories continued to evolve with the contribution of other individual scientists, such as John Tyndall and Svante Arrhenius (3). However, it was not until 1988 that the first internationally recognized organization was established to address the issue of climate change. Following a series of alarming weather events, the Intergovernmental Panel on Climate Change (IPCC) was established by the United Nations Environment Programme and the World Meteorological Organization due to concerns about the potential for global warming (4).

Global warming is primarily caused by anthropogenic activity, which releases greenhouse gases such as carbon dioxide and Methane into the atmosphere. These gases act as a heat blanket, covering the Earth's surface and making it warmer than it should be (5). The burning of coal is the largest source of carbon dioxide emissions, such that “the increase in atmospheric CO_2_ in recent decades represents about half of the emissions from fossil fuels and changes in tropical land use” (6). The remaining CO_2_ in the atmosphere is absorbed by the ocean, terrestrial biosphere, and soils (6). The primary natural source of methane release is from microbial decay of organic matter in wetlands. Anthropogenic sources account for twice as much methane as natural sources and include rice cultivation, livestock, bacterial decay in landfills, and the leakage of methane during the mining of fossil fuels (6).

While human activities are the primary drivers of global warming, some argue that natural factors, such as solar activity, volcanic eruptions, and changes in ocean circulation, also play a role (7). Thermohaline circulation, for example, helps distribute heat across the planet through the ocean's large heat capacity and its ability to transport heat efficiently (8). Solar activity causes heating in the lower atmosphere, resulting from changes in the amount of sunlight and alterations in the upper atmosphere's chemical composition due to shifts in the sun's UV rays (9). Lastly, volcanic eruptions are also a source of global warming due to the ejection of sulfur gases into the atmosphere, which are oxidized to aerosols. Aerosols spread and produce chemical and radiative effects, resulting in changes in the climate system (10). The global warming effects of aerosols include increasing cloud brightness due to increased sunlight reflection and increasing cloud coverage, as the aerosols inhibit rainfall (6).

In conclusion, global warming is a result of a combination of human activities and natural variations. “Modeling experiments suggest that anthropogenic activities dominate the warming of the 20th century, while natural forcings, such as volcanic and solar activities, are the principal drivers of climate change over the past millennium” (7).

The rate of global warming has been approximately 0.06°C per decade since 1850. However, since 1982, the rate of warming has increased to 0.20°C per decade (11).

### 1.2 Diseases affected by global warming/climate change

Numerous diseases are linked to climate change. A recent article by Mora et al. entitled “Over half of known human pathogenic diseases can be aggravated by climate change” (12) reveals that 277 diseases can worsen due to climatic hazards resulting from greenhouse gas emissions. Climatic hazards have at some point aggravated 58% of infectious diseases confronted by humanity worldwide. Additionally, this article identifies over 1,000 pathways through which various climatic hazards have contributed to disease outbreaks, primarily due to the diversity of pathogens. The significant number of pathogenic diseases and transmission pathways underscores the severe threat that climate change poses to human health, highlighting the urgent need for decisive action to reduce greenhouse gas (GHG) emissions.

Furthermore, the focus of this study is on diseases that are epidemics in nature, which are outbreaks that are not typically present in a region or population. Still, when they occur, they affect many individuals within a short period, suddenly and widely. The most prevalent diseases in the epidemic category that are driven by climate change are Dengue, Malaria, Yellow Fever, Influenza, HIV, and Monkeypox. Equally important are other infectious diseases, such as the viral diseases Zika, Ebola, and SARS-CoV-2, as well as bacterial Lyme disease and hard-tick relapsing fever (HTRF), among many others. However, many of these diseases are classified as endemic or pandemic, and their direct correlation with climate change must be analyzed with the evaluation of their host carriers—specifically, a particular species of mosquitoes, fruit bats, and blacklegged ticks. These categories of disease will not be analyzed in this study.

#### 1.2.1 Dengue

Rising global temperatures, particularly in warmer climates, influence dengue as they promote the growth and reproduction of *Aedes* mosquitoes, the primary vectors responsible for spreading the disease (13). “Monthly increases of 1°C in temperature increased the population risk of dengue by 1.95 times” (13). Dengue is highly concentrated in tropical areas and is therefore strongly correlated with temperature and the rainy season (13). In 1950, the number of global cases was approximately 908 (14), which increased to 250,000 annual cases between 1970 and 1980 (15). In 2016, a total of 291,964 cases were globally reported (16). As of 2024, there are over 7.6 million cases of Dengue globally (17).

#### 1.2.2 Malaria

Malaria is also a mosquito-borne disease found in warmer climates transmitted from the female *Anopheles* mosquito to humans. “Each 2°C–3°C increase in ambient environmental temperature increases malaria transmission by 3%–5%” (18). *Anopheles* mosquitoes thrive in warmer climates at lower altitudes, as their reproductive phases are shortened and their feeding frequencies are increased (18). Additionally, heavy precipitation is favorable for Anopheles living conditions, as in dry conditions, these mosquitoes will desiccate (19). *Anopheles* mosquitoes are at their most reproductive when a rainy season has just come to an end, as the water level decreases and water pools in areas with deformed terrain become prime for breeding (19). Global deaths caused by Malaria increased from 995,000 in 1980 to 1,817,000 in 2004 (20). As of 2021, there were 247 million cases of malaria globally (21).

#### 1.2.3 Yellow fever

Yellow fever is a viral hemorrhagic fever transmitted by infected *A. Aegyptus* mosquitoes (22). There is strong evidence that the predicted global warming will alter the international distribution of *A. Aegyptus* and increase the risk of the disease globally (23). Factors that influence and improve the occurrence of Yellow Fever include increased rainfall, higher air humidity and temperature, as well as non-primate human species, which serve as hosts for this disease (24). The mosquitoes carrying this disease have a peak population during a rainy summer, as increased rainfall and temperature (24) contribute to their proliferation. As of 1975, only 168 cases were reported in South America (25). In 2000, the global incidence rate of Yellow Fever was 0.4 per 1,000,000 total population (with 699 total cases reported in 2000) (26). In 2021, there were an estimated 86,509 cases reported globally (27).

#### 1.2.4 Influenza

Influenza is an airborne, transmitted viral disease (28) that is strongly influenced by absolute humidity, which in turn is affected by environmental temperature (29). At low temperatures, drier conditions promote the spread of the influenza virus (29). This occurs because cold temperatures make the viral envelope more fragile, and dry air helps the virus remain stable, allowing it to stay airborne for more extended periods and increasing its ability to spread (29). On the other hand, at high temperatures, higher humidity levels are more favorable for the virus's survival (29). In warm conditions, the virus is more likely to dry out; however, high humidity helps prevent this, keeping the virus viable for longer periods and facilitating its spread (29). In 1950, a total of 164,352 patients were diagnosed with influenza in the United States (30). As of 2010, the annual rate of fatalities due to Influenza was∼1.4–16.7 deaths per 100,000 persons (31). Currently, as of 2024, Influenza affects approximately 8% of adults and 25% of children annually (32).

#### 1.2.5 HIV

HIV is a viral infection caused by the human immunodeficiency virus and is spread by exposure to blood or other bodily fluids through sexual and mother-to-infant contact (33). Climate change has been linked to HIV transmission through its effects on public health, including increased food insecurity and greater human migration or climate refugees (34). Food insecurity is related to climate change as it increases substance use, resulting in a driving force for sexually risky behaviors (34). The chain of events begins with climate change-induced land degradation, followed by a reduction in food production. Then, those living in poverty may resort to desperate measures to seek food, possibly including transactional or forced sex, which raises the risk of HIV transmission (35). The first known case of HIV was reported in 1959 in Congo (36). In 2000, the total number of global cases in December 2000 was 36.1 million (37). As of 2023, there were an estimated 39.9 million global cases (38).

#### 1.2.6 Monkeypox

Monkeypox is a viral disease caused by a pox virus, a member of the genus Orthopoxvirus (39). Climate change, resulting in a warmer climate, is predicted to lead to changes in the geographic ranges at risk of Monkeypox, caused by direct climate effects on the pathogen or indirect effects on species that harbor the pathogen or vector species (40). Warmer temperatures are found to be much more apt for human MPX (40). “Specifically, for each 1°C increase in the heat index (HI), daily cases increased by 7.7%” (41). Conversely, an increase in wind speed has been found to decrease monkeypox cases (41). The first case of monkeypox was diagnosed in 1970 in a 9-month-old African baby (42). In 2007, the average incidence rate worldwide was 5.53 per 10,000 individuals (43). As of 2024, over 100,000 confirmed cases had been reported by 120 countries worldwide (44). Table 1 summarizes the climate-related incident rate of epidemics over the last 75 years.

**Table 1:** Overview of the climate-affected incident rate of epidemics during the last 75 years.

Year	Epidemy	Ref
Dengue		
1950	1950–1959, the number of global cases was approximately 908	(14)
	First epidemic in Jamaica (1963) with 1,500 cases, spread to Puerto Rico with 27,000 cases and Venezuela with 10,000 cases	(45)
1975	1970–1980, the incidence rate was ∼250,000 cases annually	(15)
	Dengue outbreak Dengue in Cuba in 1981, with 34,000 documented cases	(46)
2000	2016, there was a total of 291,964 reported cases	(16)
	2000–2011, over 860,000 cases were reported in Thailand	(47)
2024	There have been over 7.6 million cases, including 3.4 million confirmed cases	(17)
	In 2019, the Oceania region had incidence rates of 3,173.48 per 100,000 population	(48)
>2024	According to the National Library of Medicine, a model predicts that the number of populations with Dengue will increase by 2.25 billion from 2015 to 2080	(49)
Malaria		
1950	No global incidence rates	
	In the early 1950s, an epidemic occurred in China, with an incidence rate of more than 10,000 cases per 100,000 people per year	(50)
1975	Global deaths due to Malaria increased to 995,000 in 1980	(20)
	The incidence rate dropped in Guangzhou to 37.58/100,000 in 1966	(50)
2000	The malaria case incidence rate declined from 82.3 per 1,000 in 2000 to 57.2 in 2019	(21)
	By 1999, the incidence of malaria dropped to 2.47/100,000 in Guangzhou, China	(50)
2024	There were an estimated 247 million cases of malaria globally in 2021	(21)
	2022, Africa had 233 million Malaria cases	(51)
>2024	WHO plans on reducing malaria case incidence by at least 90% by 2030	(51)
Yellow Fever		
1950	No global incidence rate found—cases high in the America's	(52)
	Annual reported incidence varied in Americas between 50 and 300 annual cases	(53)
1975	1975, 168 cases were reported in five countries of South America: Bolivia, 151 cases; Brazil, 1 case; Colombia, 12 cases; Ecuador, 3 cases; and Peru, 1 case	(25)
	Bolivia had up to 151 cases annually between 1965 and 1984, with an annual mean of 34.6 cases	(54)
2000	The global incidence rate is 0.4 per 1,000,000 total population	(26)
	A total of 692 cases were reported from 1965 to 1984	(54)
2024	In 2021, an estimated 86,509 cases were reported globally	(27)
	2024, 7 confirmed cases were reported in the Americas	(55)
>2024	WHO estimates 200,000 annual cases of YF with up to 30,000 deaths	(56)
Influenza		
1950	No global incidence rates found	
	1950, 164,352 patients were diagnosed with influenza in the United States	(30)
1975	No global incidence rates found	
	1975–1976, 2,481 cases were reported by WHO in 39 states throughout the US.	(57)
2000	No global incidence rate found, outbreaks in America's, Asia, and Europe	(31)
	2010, the annual rate of fatalities in the US was ∼1.4 to 16.7 deaths per 100,000 persons.	(31)
2024	No global incidence rates found; currently affects approximately 8% of adults and 25% of children annually	(32)
	2023–2024, there were 351,460 cases in the United States	(58)
>2024	According to the WHO, there are ∼1 billion cases of seasonal influenza per year.	(57)
HIV		
1950	No global incidence rate found, 1959—the first known case of HIV was reported in Congo	(36)
	No incidence rates were found for the region—the earliest cases of HIV in the U.S. are linked to instances in Haiti	(59)
1975	No global incidence rate found	
	1980's, the incidence rate reached approximately 130,000 cases per year in the United States	(60)
2000	The total number of global cases in December 2000 was 36.1 million	(37)
	In 2006, the estimated incidence rate was 22.8 per 100,000 population in the U.S	(60)
2024	2023, there was an estimated 39.9 million global cases	(38)
	2022, there was an estimated 31,800 cases in the U.S	(61)
>2024	It is expected that by 2025, 95% of people living with HIV will know their status	(38)
Monkeypox		
1950	No global incidence rates found	
	No region incidence rates found	
1975	No global incidence rates found- first case of monkeypox diagnosed in 1970 in a 9-month-old African baby	(42)
	48 confirmed cases in six African Countries: DRC, Cameroon, Cote D'Ivoire, Liberia, Nigerai and Sierra Leone	(42)
2000	In 2007, the average incidence rate across the world was 5.53 per 10,000 individuals	(43)
	In 2003, there was an Outbreak of 47 cases in the U.S., including confirmed and probable cases	(42)
2024	Over 100,000 confirmed cases reported by 120 countries globally	(44)
	The average incidence rate in the U.S was 14.3 cases per 100,000 individuals in 2022	(62)
>2024	According to the WHO, an outbreak of monkeypox has been ongoing in the DRC since 2022, with the potential to spread to neighboring countries and trigger a public health emergency of international concern	(63)

## 2 Discussion

### 2.1 Organizations responding to climate-related diseases to decelerate the incidence rates

#### 2.1.1 Dengue

In response to the emergence of dengue as an epidemic in more than 100 countries, in 2024, the World Health Organization launched the Global Strategic Preparedness, Readiness and Response Plan (SPRP) (64). Their aim is, and has been, to coordinate a global response. Their plan involves 5 primary tenets: (1) Emergency coordination (setting up a team with leaders to handle the situation), (2) Collaborative surveillance: creating tools to quickly spot and control outbreaks of Dengue (within the lab), (3) Community protection (working closely with communities to share information and prevention strategies), (4) Safe and Scalable Care (ensuring health services are equipped to provide adequate care to patients with dengue, and (5) Access to countermeasures (continuing ongoing research to find better treatments and vaccines) (64). Due to the rapid global spread of dengue, the WHO has also employed a risk mapping strategy to assess which countries are currently at risk of dengue and which countries are vulnerable to the onset of dengue transmission (64). Their objective in using the risk map was to pinpoint countries where preparedness and response interventions were required, and in such cases, these countries would be registered for increased surveillance (64). WHO has also adopted the approach of using vector control strategies, including removing breeding sites and employing chemical agents to kill the larvae of Aedes Mosquitoes (64). The World Health Organization is aware of the role that climate change plays in the increase of dengue cases and is therefore adjusting dengue control methods based on different levels of risk, considering how changing climate patterns may impact the location of mosquito breeding sites (64). The SPRP is in the stages of developing an efficient diagnostic test, efficient treatments options and a vaccine for prevention (64). There have been dengue vaccines released in the past, such as Dengvaxia and Qdenga, and another more efficient dengue vaccine is currently in the middle of clinical development (64). The WHO has recommended the dengue vaccine for routine immunization programs in areas with high transmission rates and where dengue is a major public health concern, specifically for children aged 9–16 years (64).

#### 2.1.2 Malaria

As Malaria emerges as a more prevalent disease globally, the World Health Organization administered the Global Technical Strategy for Malaria 2016–2030 (65). Their objective is to reduce the global malaria rates by 90% by 2030 (65). WHO plans to eliminate malaria from countries in which malaria was prevalent in 2015 in at least 20 of such countries by 2025, and at least 35 of those countries by 2030 (65). The malaria eradication strategy is based on five key principles: tailoring approaches to local needs, strong leadership and community involvement, enhanced data tracking, equitable access to health services, and innovation in new tools to accelerate progress towards elimination (65). WHO also emphasizes the importance of vector control strategies in eradicating malaria, utilizing tools such as long-lasting insecticidal nets and indoor spraying (65). Research is needed to figure out where and how targeting the parasite reservoir can help reduce malaria (65).

#### 2.1.3 Yellow fever

The long-term (2017–2026) global strategy of the World Health Organization to “Eliminate Yellow Fever Epidemics” (EYE) focuses on three main goals: “(1) protecting at-risk populations, (2) preventing international spread, and (3) containing outbreaks quickly” (66). The strategy includes recommendations of vaccination, building resistant cities that are ready for emergencies and making sure international health rules are followed more effectively (66). The EYE strategy exclusively targets countries that are at the highest level of risk for a yellow fever outbreak (66). In the next ten years, vaccine makers are expected to provide 1.38 billion doses (66). Also, the WHO has adopted the Integrated Disease Surveillance and Response (IDSR) program to obtain a data count of suspected, probable, and confirmed yellow fever cases (66). By the end of 2025, WHO suspects that all high-risk countries in Africa can now test and confirm yellow fever and by the end of 2026, all high-risk countries will have completed national vaccination campaigns (66).

#### 2.1.4 Influenza

In June 2024, the Center for Disease Control and Prevention (CDC) launched the Global Influenza Program (67). CDC is teamed up with global partners to improve their response to Influenza threats (67). Their programs core tenets are to work with partners to improve flu surveillance, develop pandemic plans (and prevention plans), support research projects, and expand flu vaccination programs (67). As well, CDC is partnered with the World Health Organization to monitor seasonal flu, detect novel flu and work on a vaccine (67). The WHO predicts that by 2030, we will have better global tools, including a specific plan with more research devoted to preventing, detecting, controlling, and treating influenza, along with stronger country capacities, where each country will have its own customized influenza program, ensuring global health preparedness (68).

#### 2.1.5 HIV

By the end of 2025, Canada's global target is to have 95% of people living with HIV diagnosed, and 95% of those diagnosed on treatment, along with 95% of those on treatment responding positively to the treatment with reduced viral load of HIV (69) The Global Health HIV Prevention Clinic (GPC) also launched the HIV prevention strategy 2025 in which they predict to have fewer than 370,000 annual new cases of HIV by the end of 2025 (70). The road map that GPC created includes 10 steps: (1) conduct assessments and gather data on HIV prevention programs; (2) adopt a precise prevention approach focused on target populations; (3) identify the country's financial investment needs for adequate HIV responses; (4) strengthen leadership for HIV prevention by promoting collaboration, oversight, and management; (5) boost community-led HIV prevention services; (6) eradicate social and legal barriers to HIV prevention services for target populations. (7) integrate HIV prevention into key services to improve outcomes; (8) create systems to introduce new HIV prevention tools and innovations quickly; (9) set up real-time monitoring systems for prevention programs with regular updates; and (10) improve accountability for everyone involved in HIV prevention progress (70). Their actions focus on combination prevention for key groups, like adolescent girls, young women, men, and boys in high-risk areas, promoting condoms and lubricants and expanding access to antiretroviral prevention, including PrEP (Pre-exposure Prophylaxis) (70).

#### 2.1.6 Monkeypox

As of 2024, the World Health Organization has launched the Global Strategic Preparedness and Response Plan to combat the outbreak of monkeypox (71). Their plan focuses on implementing robust surveillance, prevention, and response strategies; enhancing access to medical tools such as tests and vaccines; reducing animal-to-human transmission; and engaging communities in outbreak control (71). The WHO is actively working on an efficient vaccine for individuals who are the most vulnerable, specifically healthcare workers in contact with recent cases (71). Globally, the focus is on strong leadership, timely guidance, and providing medical tools to high-risk groups in affected countries (71). In Africa, WHO Regional Office (AFRO) and Africa CDC will lead the mpox response, using a shared plan and budget (71). At the national and local levels, health authorities will adjust strategies based on the current situation (71). Currently, there is no specific antiviral treatment for Monkeypox, which is a growing global concern (72).

### 2.2 The World Health Organization's response to climate-related diseases

The World Health Organization recognizes the increasing risk that global warming has on climate-related epidemics (73). They are aware that an increase in temperature and precipitation boosts the spread of vector-borne diseases and that “without preventative actions, deaths from such diseases, currently over 700,000 annually, may rise” (73). Therefore, in response to this WHO has created an approach to avert health impacts due to climate change (73). WHO's response is based on three main objectives. The first is to “promote actions that both reduce carbon emissions and improve health” (73). This centers health as the primary concern of climate change, ensuring that actions benefit both the environment and health, and utilizes the health community to advocate for policies that protect both (73). Secondly, WHO is aiming to build better and more “climate-resilient and environmentally sustainable health systems”(73). This includes helping health systems adopt more affordable, environmentally friendly solutions and ensuring that health investments support sustainability and climate adaptation, including training healthcare workers (73). The third objective of the WHO is to “protect health from the wide range of climate change” (73). This includes identifying climate-related health risks, enhancing our systems' capacity to respond to such risks, and providing funding for health systems to adapt to climate change (73). “WHO strives to embed climate change in health priorities, similar to those of United Health Care, and target carbon neutrality by 2030” (73).

### 2.3 New discipline in epidemiology that focuses exclusively on climate change-related prediction for future pandemics

“Climate epidemiology” appears to be a growing research discipline, driven in response to the perceived public health risks associated with climate change. In recent decades, greenhouse gases have been the primary driver of climate change (74). Furthermore, multiple studies have demonstrated that climate change influences disease transmission, although the exact nature of the causal impact is not always known (75). As global warming continues to accelerate, climate-related illnesses, such as vector-borne diseases, are expected to become an increasing threat to public health (76).

Climate epidemiology involves collaboration between epidemiologists and climate scientists to understand the connections between climate change and the emergence of infectious diseases, including the prediction of future pandemics (74). It does so by examining how climate change (including rising air, land, and sea temperatures, altered precipitation patterns, extreme weather events, and shifts in ecosystems) may affect the transmission and spread of infectious diseases (74). More specifically, scientists in this field aim to determine how the environment influences disease vectors (such as mosquitoes or ticks), how shifts in ecosystems may impact animal hosts, and how human migration or population displacement due to climate events can introduce new pathogens into different regions (74).

The ultimate goal of climate epidemiology is to provide insight into the causation and effects of disease spread through climate modeling, in a way that can be used to help predict potential issues and develop preventive strategies.

### 2.4 Climate-driven model of diseases

Globally, nations such as the US, Europe, and Australia are attempting to preserve the environment to combat the transmission of dengue (77). By clearing dirty environments, this makes the habitat less favorable for mosquitoes to live in, as they prefer areas with high levels of filth. Campaigns to sanitize bodies of water are being initiated by voluntary organizations such as the India Peace Youth Movement, the American Federation of Students, and the National Youth Society (77). Another strategy that Japan has adopted is spreading educational knowledge to students in preventing dengue through environmentally sustainable practices (77).

In 2021, two organizations—the World Health Organization's Strategic Advisory Group on Malaria Eradication and the Lancet Commission on Malaria Eradication—proposed strategies to combat global malaria, which is influenced by a climate-driven model (78). The first strategy outlined involves using accurate risk mapping to identify malaria transmission hotspots, specifically areas where climate conditions make the region more susceptible to transmission (78). Their other strategy is to research and find effective insecticides, which are dependent on the fact that certain insecticides are less effective in hotter climates (78). These organizations are also incorporating climate considerations into their malaria control efforts, such as investing in surveillance systems that monitor both climate and malaria (78). This is essential to understand and implement knowledge regarding the complex relationship between climate and malaria transmission.

### 2.5 From the patient's point of view—how do non-medical/health professionals contribute to minimizing the spread of climate-related diseases?

From the patient's perspective, non-medical and non-healthcare professionals can play a vital role in minimizing the spread of climate-related diseases and preventing new epidemics by collaborating to reduce public vulnerability. The vulnerability of a population or region to the impacts of climate change is a function of (79).

• Exposure to climate hazards
• Sensitivity to those impacts
• Adaptive capacity

#### 2.5.1 Exposure

Reducing exposure to climate hazards can be achieved by preventing them in the first place, which starts with increased awareness and education regarding the threat of climate change. A recent study conducted on 2,273 participants in more than 30 countries revealed a strong correlation between people's belief in climate change and their willingness to make behavioral changes to mitigate it (80). Furthermore, according to the American Journal of Health, only one in five Americans reports having a strong understanding of climate change (81). Moreover, roughly half of Americans express distrust of related media coverage.

Moving beyond essential awareness, citizens can further mitigate the risk of climate change exposure by supporting government policy change, as well as reducing their carbon footprint (82). Although broad public support is not always a prerequisite for implementing policies designed to reduce greenhouse gases, it has been demonstrated to facilitate the adoption and implementation of robust policies (82).

On a personal level, individual citizens can take simple steps to reduce their carbon footprint, such as using public transportation, reducing waste, conserving water, and eating a more plant-based diet (83). Environmental studies have shown that daily household consumption is the primary contributor to greenhouse gases, accounting for two-thirds of global carbon emissions (83). When undertaken collectively, individual actions can contribute to the broader effort of mitigating the long-term impact of climate change.

#### 2.5.2 Sensitivity

Sensitivity to the societal impacts of current climate change can be influenced by a multitude of factors directly related to health and well-being, including socioeconomic status, biology and genetic endowment, access to health services, gender, and personal health practices (79). Similar to mitigating the risk of exposure, individuals can help lower sensitivity to climate change-driven health threats by raising personal awareness and supporting community initiatives (79).

One type of public initiative to reduce climate change sensitivity is creating more resilient infrastructure to reduce the impact of climate change, such as “the designing of flood-resistant homes, improving waste management systems, or building more green spaces can limit the conditions that foster disease outbreaks, particularly in vulnerable populations” (84). Unfortunately, the current reality is that longer-term climate-related risks are not always taken into account in the shorter-term profit-focused public and private investment decisions. As a result, consequently, data shows that large amounts of capital continue to flow into hazard-prone areas (84).

Another form of public initiative to reduce climate change sensitivity is to ensure disaster preparedness. Providing immediate aid, setting up shelter, ensuring clean water, and managing sanitation can help reduce the risk of secondary diseases following a natural disaster.

#### 2.5.3 Adaptive capacity

Adaptive capacity is “the potential or ability of a system, region, or community to adapt to the effects or impacts of climate change” (85). Communities that have established high adaptive capacity, as evidenced by comprehensive plans and responses, have been shown to provide citizens with a better ability to protect their own health (79). Human adaptation is crucial in mitigating the socio-economic impacts of climate change on ecosystems and populations (86). Non-medical citizens can also strengthen these measures through education and support.

A prime example of adaptive behavior is the water management initiatives under way in the UK (87). UK researchers are using climate change factors to predict future alterations in rainfall and river flow. These estimates are then incorporated into the long-term plans of water utility companies to ensure the sustainability of water supply (87).

As climate change influences the spread of vector-borne diseases such as malaria, dengue, and Zika, personal adaptive health measures that citizens can employ include limiting exposure (using insect repellent and wearing protective clothing when outdoors), eliminating areas of standing water, and using bed nets when sleeping (88). As an example of the effectiveness of one of these solutions, the World Health Organization (WHO) estimated that between 2000 and 2015, the annual incidence of malaria cases fell by 37% and that the malaria mortality rate fell by 60% (88) These reductions are believed to have been attained, in large part, due to the increase in the use of insecticide-treated bed nets and access to treatment. However, the WHO also indicated that only about 68% of individuals at risk of malaria slept under an insecticide-treated bed net in 2015, so the reduction in malaria could have been even more significant with improved precautions.

For viral-based or waterborne diseases, personal adaptive measures could include regular hand washing, boiling potentially unsafe water, and/or avoiding drinking from unknown sources. The U.S. Centers for Disease Control and Prevention has cited hand washing as the single most effective way to prevent the transmission of disease (89).

### 2.6 Outbreaks vs. urbanization/pollution/increase in population density and public health policies

#### 2.6.1 Dengue

Although climate patterns highly influence the transmission of dengue, it has been shown that dengue cases are not always consistently distributed in cities where climate is considered uniform (90). Therefore, there are other confounding factors at play in the outbreak of dengue (90). Such factors include socioeconomic statuses (SES), population growth, mass human movements, and urbanization (90). A study done in Nouméa during two major dengue epidemics (2008–2009 and 2012–2013) reveals that the neighborhoods with lower socioeconomic statuses were correlated with higher dengue incidence rates (90). Rapid urbanization has been found to play a large role in the outbreak of dengue, as it enables easier distribution of the *Aedes* population (91). Urban developments are creating more suitable habitats for *Aedes* mosquitoes, such as highly concentrated areas of human density, which provide more opportunities for blood feeding, and increased areas of moisture, like flowerpots (91). Ultimately, it is not only climate change that plays a role in the increase of dengue, but multiple variables are at play in this disease's transmission.

#### 2.6.2 Malaria

For malaria, it appears that areas of large human densities contribute to a decline in the spread of malaria (92). This is because urbanization and anthropogenic factors create a less suitable breeding ground for *Anopheles* mosquito species (92). These anthropogenic variables include fewer water surfaces for breeding and natural surfaces covered with infrastructure (92). Additionally, urbanization contributes to increased pollution, which negatively impacts breeding sites for *Anopheles* species (92). Other anthropogenic factors that contribute to the spread of malaria include agriculture (93). For instance, when farmers construct irrigation channels, this appears to create suitable mosquito breeding sites (93). Lastly, low socioeconomic factors also contribute to an increase in malaria cases, as those with lower socioeconomic statuses will have less access to health care and risk prevention (94).

#### 2.6.3 Yellow fever

Other confounding factors that contribute to an increase in Yellow Fever outbreaks include social, ecological, and behavioural factors (95). Such factors include the movement of human populations into forest areas and coastal zones, where there is an increased number of breeding sites for *Aedes aegypti* mosquitoes (95). The number of non-vaccinated human populations also plays a role in the spread of Yellow Fever (95). It appears that urbanization has caused an increase in Yellow Fever outbreaks (96). According to WHO, an outbreak in Angola in 2016 was due to a large urban outbreak in a transportation center, which showed how Yellow Fever can rapidly spread to foreign nations (96). In Africa, growing and increasingly populated cities (increasing by 4% annually) appear to play a role in the increase of Yellow Fever, as unvaccinated populations are exposed to drinking water in large open containers—favourable breeding sites for *Aedes aegypti* mosquitoes (97). Fortunately, public health initiatives like Oswaldo Cruz's campaign against the mosquito vector *Aedes aegypti* drastically reduced Yellow Fever cases in Rio de Janeiro (95). In the early 1940s, both vaccination campaigns and the eradication of *Aedes aegypti* significantly contributed to the elimination of urban Yellow Fever transmission in Brazil and across the Americas (95).

#### 2.6.4 Influenza

Aside from climate change contributing to the spread of influenza, other impacting factors like urbanization have a major effect on the intensity of influenza epidemics (98). Urbanization shapes the severity of influenza's transmission patterns due to increased human population densities, which increase human contact and population mobility (98). It appears that cities are the principal areas for human influenza transmissions, with more diffuse epidemics occurring in more populated cities, as there are higher rates of personal contact (99). It also appears that in smaller cities, surges of influenza outbreaks last shorter periods of time (99). Regarding the public health interventions that were set in place during the peak of COVID-19, this decreased the cases of influenza from 71.9 per 1,000 people to 49.8 per 1,000 people (100). Such public health interventions included remaining at home when experiencing respiratory symptoms, wearing masks in public spaces, and hand sanitization (100).

#### 2.6.5 HIV

A strongly contributing factor to the prevalence of HIV outbreaks is in-migration into large cities (101). Migration increases opportunities for contact between humans, growing sexual networks from different areas, and ultimately increasing HIV's transmission rate (101). According to the article “*HIV/AIDS and Urbanization”*, “urban levels of HIV infection are typically four to ten times those of rural areas” (102). Public health policies have helped reduce HIV cases in certain parts of the world, particularly in Africa, where the President's Emergency Plan for AIDS Relief was employed (103). It is thought that through advances in treatment, the HIV epidemic in the United States could be quickly ended (103).

#### 2.6.6 Monkeypox

Although the chances of Monkeypox outbreaks tend to increase in warmer climates, there are other variables that play a role in its transmission. The surge of Monkeypox cases in Nigeria in 2017 was driven by a combination of factors, including population growth and unvaccinated residents (104). Two key factors contributing to the outbreak were increased exposure of humans to forest animals due to deforestation and population migration, and declining vaccination immunity since the 1970s (104). Additionally, globalization has increased the likelihood that Monkeypox will spread around the world, as humans are more connected, giving way to quicker disease transmission (104).

Generally, there appears to be a strong correlation between high infection rates and urban areas (denser populations) (105). Another variable that has appeared to contribute to an increase in Monkeypox cases includes environmental pollution, such that air pollutants like CO and NO₂ have a positive association with daily Monkeypox cases (106).

### 2.7 Implementation effects—the vaccination coverage vs. case reduction rate

#### 2.7.1 Dengue

##### 2.7.1.1 TAK-003 vaccine (Asia & Latin America)

In a substantial Phase 3 trial, TAK-003 showed an overall efficacy of 80.9% in preventing virologically confirmed dengue, reporting 0.5 cases per 100 person-years in the vaccine group vs. 2.5 cases per 100 person-years in the placebo group. The vaccine group also experienced a 95.4% reduction in hospitalizations compared to the placebo (107–109). In Sri Lanka, TAK-003 led to a projected 69.1% decrease in dengue cases and a 72.7% decline in hospitalizations during an outbreak. With improved coverage and accelerated rollout, these reductions rose to over 80% (110).

##### 2.7.1.2 CYD-TDV (dengvaxia) vaccine

In Latin America, a significant phase 3 trial reported 60.8% efficacy against symptomatic dengue and 80.3% efficacy against hospitalizations, along with a 95.5% reduction in severe cases among vaccinated children aged 9–16 (111). Mass vaccination campaigns in Brazil's Paraná State resulted in an overall effectiveness of 21.3% in reducing symptomatic dengue cases, with a 71% decrease noted among individuals with a previous dengue history. For those without such a history, effectiveness was lower at 12% (112). A 6-year cohort study conducted in southern Brazil revealed a 33.7% reduction in probable dengue cases and a 20.1% reduction in laboratory-confirmed cases, with a notable increase in effectiveness for the DENV-1 and DENV-4 serotypes (113).

Additionally, modelling studies have shown that in areas with high transmission, vaccinating 9-year-olds with an 80% coverage rate could lead to a remarkable reduction in the dengue burden by 13%–25% over the next 30 years. In moderate-to-high endemicity areas, the reductions were found to range from 6% to 25% (108). In Yucatán, Mexico, models are predicting an impressive decrease of up to 80% in annual dengue incidence within just 5 years if a durable vaccine is implemented (109).

Key factors influencing the reduction rates of dengue include serostatus—the presence or absence of specific antibodies in a person's blood serum, as identified by a serological test—and virus serotype, which refers to a serologically distinguishable strain of a virus. Considering the efficacy of vaccines based on serostatus, studies have indicated that vaccination is more effective in individuals with prior dengue exposure (i.e., seropositive). Simultaneously, some cases have shown an increased risk or reduced effectiveness in seronegative individuals (112, 114, 115). The efficacy of vaccines varies by dengue virus serotype, with lower effectiveness against DENV-2 and higher effectiveness against DENV-3 and DENV-4 (111, 113, 116).

Dengue vaccination has made a remarkable impact by significantly reducing the number of dengue cases and hospitalizations, especially among high-transmission, seropositive populations. The reduction rates vary widely, ranging from 20% to over 80%, depending on the type of vaccine used, the setting, and the characteristics of the population involved. Yet, there's still more we can do to improve these outcomes! For example, achieving higher vaccination coverage and starting campaigns promptly can lead to even greater decreases in cases and hospitalizations, as highlighted in literature (109, 110). Additionally, it's vital to assess the durability of the vaccine to address the issue of waning immunity, which could lead to larger epidemics if booster doses aren't given as needed (109).

#### 2.7.2 Malaria

Malaria continues to pose a significant health challenge around the world, yet we've made remarkable strides in reducing both the number of cases and deaths, especially with the advent of new vaccines. The recent malaria vaccines, particularly RTS, S/AS01 and R21/Matrix-M, have been instrumental in significantly lowering malaria cases among children in regions that bear the highest burden. Between 2000 and 2015, global malaria incidence rates experienced an impressive drop of 37%, and mortality rates decreased by an incredible 60%. This success is largely due to the increased use of insecticide-treated nets, enhanced diagnostics, and effective antimalarial treatments—vaccines came into play later, as they weren't widely accessible during this period (117, 118). In sub-Saharan Africa, an astonishing 70% of the reduction in cases can be credited to these vital interventions (117).

The impact of malaria vaccines on the incidence rate is truly remarkable. One shining example is the RTS S/AS01 vaccine, highly recommended for children living in high-transmission areas. This vaccine has shown that it can prevent a median of 2,653 malaria cases for every 100,000 people each year, and it has helped avoid around 82,270 cases in fully vaccinated children per 100,000 (119–121). In long-term studies, the number of severe malaria cases in vaccinated children has stayed impressively low, ranging from just 0.004 to 0.007 cases per person-year, with no notable rise in cases observed 6–7 years after vaccination (122). Additionally, the newer R21/Matrix-M vaccine has proven to be effective as well, showcasing 75% efficacy at seasonal sites and 68% at standard sites, leading to a decrease of 868 malaria cases per 1,000 child-years at seasonal sites and 296 per 1,000 at standard sites over the course of 12 months (118).

It is encouraging to observe that acceptance rates for malaria vaccines are notably high in endemic countries, attaining an impressive rate of 95.3%. This indicates substantial potential for achieving a widespread impact as these vaccines become increasingly accessible. Prior to the introduction of vaccines, the decline in malaria cases was primarily attributed to vector control measures and treatment protocols. However, the advent of vaccines such as RTS, S/AS01 and R21/Matrix-M has resulted in even more significant reductions in malaria cases among children, particularly in Africa. Given such elevated vaccine acceptance rates, we are optimistic about the ongoing advancements we can achieve in the fight against malaria.

#### 2.7.3 Yellow fever

Yellow fever is a serious viral disease found in various regions of Africa and South America. The introduction and expansion of yellow fever vaccination campaigns have made a remarkable difference, leading to significant reductions in cases and deaths around the world, especially in high-risk areas. Thanks to mass vaccination, we've seen a substantial decline in the incidence of yellow fever and outbreaks.

In Africa, these mass vaccination efforts have resulted in an impressive 47% reduction in yellow fever deaths in 2018 when compared to what might have happened without the vaccine, preventing around 10,000 deaths that year alone (123). Preventive mass vaccination campaigns (PMVCs) across African provinces have brought about an incredible 86% decrease in the incidence of yellow fever outbreaks (with an incidence rate ratio of 0.14), leading to a 34% reduction in outbreaks during the study period from 2005 to 2018 (124–131). Between 2005 and 2017, vaccination activities in Africa are estimated to have prevented between 3.3 and 6.1 million deaths over the lifetimes of those vaccinated, depending on the level of herd immunity considered (127).

Since 1970, global yellow fever vaccination coverage has improved significantly, yet as of 2016, 43%–52% of individuals in risk zones still needed vaccination to reach the recommended 80% coverage threshold for preventing outbreaks (125). While vaccination has been especially effective in high-risk areas, there are still gaps in some regions, particularly in certain parts of Nigeria, the Democratic Republic of Congo, and South Sudan (128).

Overall, yellow fever vaccination campaigns have had a significant impact, reducing cases, deaths, and outbreaks, particularly in Africa. However, we must continue our efforts to close these coverage gaps and maintain high immunity levels to prevent future outbreaks.

#### 2.7.4 Influenza

Vaccines play a vital role in keeping us healthy by consistently reducing cases of influenza, hospitalizations, and severe complications. The greatest benefits are seen in children and those in high-risk groups. In the United States from 2016 to 2018, influenza vaccination helped prevent up to 46.6 cases for every 1,000 vaccinated children (ages 6 months to 8 years) and 6.9 cases for every 1,000 vaccinated adults aged 65 and older during seasons when the vaccine effectiveness ranged between 29% and 40% (132). Over in Europe during the 2022/23 season, vaccine effectiveness against influenza A varied from 27% to 44% across all ages, with children benefiting the most at 49%–77%. For influenza B, vaccine effectiveness was impressive at over 50% overall, soaring to 87%–95% in children (133). In the US during the 2019/20 season, vaccination lowered the risk of influenza-related hospitalization by 41% in adults (132). A meta-analysis reveals that vaccine effectiveness among children is 39% for reducing medical visits and an excellent 57% for cutting down hospitalizations; for the elderly, it stands at 25% for visits and 14% for hospitalizations (134, 135).

#### 2.7.5 Monkeypox

Getting vaccinated against monkeypox, especially with the JYNNEOS vaccine, plays a vital role in minimizing the spread and impact of monkeypox outbreaks across the globe. While we don't have specific numbers showing the reduction in global cases before and after vaccination, there's clear evidence that the vaccine produces strong immune responses. These responses are expected to lead to lower case numbers and a lighter disease burden.

The JYNNEOS vaccine elicits a robust antibody response against monkeypox and related orthopoxviruses, with its peak response occurring approximately 2 weeks after the second dose, regardless of whether the individual has received prior vaccination (136). Most participants continued to have orthopoxvirus-specific antibodies for up to 2 years after vaccination, indicating that this vaccine provides long-lasting immune protection (136). It also effectively generates neutralizing antibodies, which are crucial for preventing infection and reducing transmission (136). Even though we don't have specific global case reduction rates detailed before and after vaccination, the strong immune response and the long-lasting presence of antibodies suggest that the JYNNEOS vaccination is likely making a significant impact in reducing monkeypox cases and helping to manage outbreaks.

## 3 Recommendations to the public to minimize the spread of climate-related disease

To minimize the spread of climate-related diseases, it's essential to take steps that both reduce exposure to the factors driving climate change and enhance personal health resilience. Here are some general recommendations:

1. Reduce carbon emissions by adopting new skills such as using public transport or carpooling, switching to renewable energy sources, and implementing energy-efficient practices in homes and workplaces (137).
2. Protect against vector-borne diseases by employing strategies like wearing insect repellent and protective clothing when going outdoors, eliminating standing water to prevent mosquito breeding, and using bed nets when sleeping in areas where diseases are common (138).
3. Stay informed about climate-related health risks in your region and get vaccinated to diminish the risk of diseases (139, 140).

## Data Availability

The original contributions presented in the study are included in the article/Supplementary Material, further inquiries can be directed to the corresponding author.
